# Ancient introgression drives adaptation to cooler and drier mountain habitats in a cypress species complex

**DOI:** 10.1038/s42003-019-0445-z

**Published:** 2019-06-18

**Authors:** Yazhen Ma, Ji Wang, Quanjun Hu, Jialiang Li, Yongshuai Sun, Lei Zhang, Richard J. Abbott, Jianquan Liu, Kangshan Mao

**Affiliations:** 1 Key Laboratory of Bio-Resource and Eco-Environment of Ministry of Education, College of Life Sciences, State Key Laboratory of Hydraulics and Mountain River Engineering, Sichuan University, 610065 Chengdu, Sichuan P. R. China; 2 Key Laboratory of Tropical Forest Ecology, Xishuangbanna Tropical Botanical Garden, Chinese Academy of Sciences, 666303 Mengla, P. R. China; 3 School of Biology, Mitchell Building, University of St Andrews, St Andrews, Fife, KY16 9TH UK

**Keywords:** Biodiversity, Molecular ecology, Evolutionary ecology, Population genetics

## Abstract

Introgression may act as an important source of new genetic variation to facilitate the adaptation of organisms to new environments, yet how introgression might enable tree species to adapt to higher latitudes and elevations remains unclear. Applying whole-transcriptome sequencing and population genetic analyses, we present an example of ancient introgression from a cypress species (*Cupressus gigantea*) that occurs at higher latitude and elevation on the Qinghai-Tibet Plateau into a related species (*C. duclouxiana*), which has likely aided the latter species to extend its range by colonizing cooler and drier mountain habitats during postglacial periods. We show that 16 introgressed candidate adaptive loci could have played pivotal roles in response to diverse stresses experienced in a high-elevation environment. Our findings provide new insights into the evolutionary history of Qinghai-Tibet Plateau plants and the importance of introgression in the adaptation of species to climate change.

## Abstract (web summary)

Yazhen Ma et al. find evidence of an ancient introgression from cypress into a related species from the Qinghai-Tibet Plateau, which aided in the colonization of cooler and drier mountain habitats. They identify 16 introgressed loci that may provide protection against high-elevation stressors.

## Introduction

Rapid climate change is poised this century to be one of the greatest global challenges to the stability and cohesion of ecosystems^1,2^. It is argued that to survive such change, terrestrial species might migrate across land by tracking favorable conditions or persist in situ either through adaptive phenotypic plasticity^3^ and/or genetic modification caused by natural selection favoring advantageous standing genetic variation and mutations^1,4^. A further, but until recently, overlooked potential source of genetic variation that can aid adaptation to climate change is introgression from closely related species^5,6^.

Recent studies on humans, dogs, cattle, sunflowers, poplars, and oaks suggest that introgression from other species can be a very important source of new genetic variation in the adaptation of species to new environments^7–12^. Such adaptive introgression resulting from hybridization and backcrossing with another species^13–15^ has the potential to allow adaptation in recipient populations at rates considerably higher than if mutation was the only source of variation^16–18^. Though adaptive introgression has long been considered an important evolutionary mechanism^13^, with notable examples reported in both plant and animal genera^10,18–22^, most studies of adaptive introgression in plants using a genomic approach have focused on short generation species (but see refs. ^11,23^). A major challenge to detecting introgression between species with long generation times and large effective population sizes, such as trees, is to distinguish between the causes of shared polymorphisms due to incomplete lineage sorting or introgression^24,25^. Notwithstanding this difficulty, a growing number of studies have shown that introgression occurs in at least some tree genera (reviewed in refs. ^5,26,27^), particularly from local tree species into widespread or invasive ones^28^, involving genomic fragments exhibiting low rather than high intraspecific gene flow^29^. How commonly such introgression is adaptive, however, remains a topic of active research.

Adaptive genetic variation is common within tree species^30,31^ with clines reflecting local adaptation across environmental gradients^1,31^ and ecotypic variation is often recorded^4^. Moreover, candidate loci associated with adaptive changes across environmental gradients are increasingly implicated in studies of adaptive divergence in trees^31–34^. Although evidence of adaptive introgression in trees is accumulating (e.g., refs. ^35,36^), only recently has detailed evidence of it emerged from genomic analyses in model tree genera such as poplars^11,23,37^ and oaks^38^. In contrast, little is known of it occurring in conifers, partly due to their huge genome size^39^ which makes reference genome assembly difficult. Moreover, very few studies have focused on introgression across elevation gradients (but see ref. ^27^), especially in topographically complex regions, such as the Qinghai-Tibet Plateau and adjacent regions in Asia (but see ref. ^40^). Here, we examine the possible role of introgressive hybridization in the adaptation of a cypress (*Cupressus*) species to higher elevations and latitudes in the Qinghai-Tibet Plateau and neighboring areas.

*Cupressus gigantea*, which occurs along the Yarlung Tsangpo river valley in Tibet (Xizang), grows at higher altitudes (2950–3430 m.a.s.l.) relative to other species in the genus^41^, whereas *C. duclouxiana*, occupies a wider altitude range (1400–3300 m.a.s.l.), and is mainly distributed in central and northwest Yunnan, and southwest Sichuan, China^42^. Previous work has indicated a close phylogenetic relationship between these two species based on cpDNA variation^43^, and an absence of gene flow between them based on nuclear microsatellite markers^44^. Importantly, populations of *C. duclouxiana* in the northern and southern parts of its range are genetically divergent, forming two separate groups based on microsatellite variation^44^. The northern populations tend to occur at elevations and latitudes between those of southern *C. duclouxiana* and *C. gigantea* populations, although their habitat is similar to that of *C. gigantea*. Thus, both *C. gigantea* and northern populations of *C. duclouxiana* occur in mountain valleys that are cooler and drier than the habitat of southern populations of *C. duclouxiana*. The distribution of *C. duclouxiana* is also notably more strongly influenced by human activities than *C. gigantea*, with relict populations found around temples and in remote areas. Here, we tested whether adaptive introgression from *C. gigantea* to northern *C. duclouxiana* enabled the latter to colonize mountain habitats.

We analysed RNA-sequences from leaf samples representing both *Cupressus* species to address the following questions: What is the genetic affinity of northern to southern populations of *C. duclouxiana* and to populations of *C. gigantea*? What is the level and direction of gene flow between the different genetic groups detected? What role might adaptive introgression have played in shaping the distribution pattern of genetic variation in this complex, enabling *C. duclouxiana* to grow over a wide range of elevations? Our results suggest that both *C. duclouxiana* and *C. gigantea* are monophyletic; however, genetic introgression from *C. gigantea* to northern *C. duclouxiana* is supported by multiple lines of evidence. We detected 1285 loci likely to be introgressed from the former to the latter, with 16 of these identified as candidate adaptive loci that might play pivotal roles in response to diverse stresses in high-elevation environments. These loci were positively selected in *C. gigantea* and northern *C. duclouxiana*, but not in southern *C. duclouxiana*. This introgression most likely occurred during the last glacial maximum (ca. 0.022 million years ago) or during earlier glacial periods, and contributed to the adaptation of northern *C. duclouxiana* to cooler and drier conditions in mountain habitats.

## Results

### Habitat differentiation

**Fig. 1:**
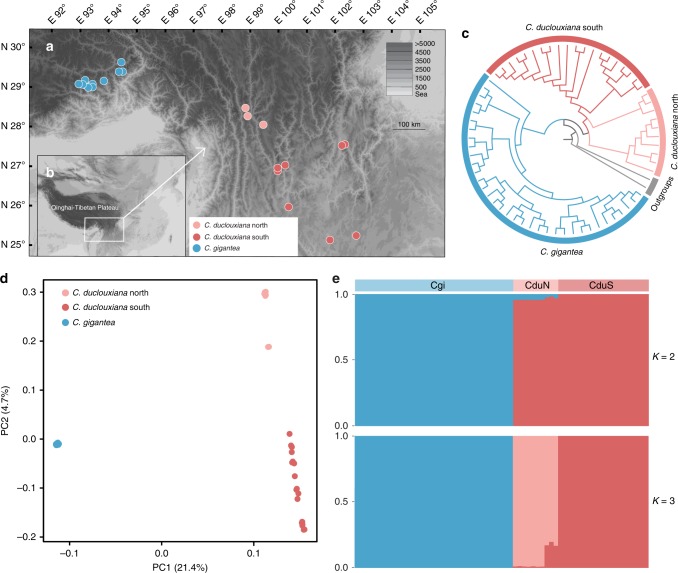
Phylogenetic and population genetic analyses of *C. gigantea* and *C. duclouxiana* based on SNP variation. **a** Map showing the geographic distribution of sampling locations for *C. gigantea* and *C. duclouxiana* populations, with **b** the location of the Qinghai-Tibet Plateau. **c** A maximum likelihood phylogenetic tree. **d** Principal component analysis (PCA) plot of the first two components. **e** Population structure plots with *K* = 2 and 3. The *x*-axis shows the different individuals of *C. gigantea* (Cgi) and of *C. duclouxiana* northern (CduN) and southern (CduS) populations; the *y*-axis quantifies the proportion of an individual’s variation from inferred ancestral populations

Leaf samples were collected from a total of 65 individuals comprising 30 *Cupressus duclouxiana* trees (from 11 populations) and 35* C. gigantea* trees (from 12 populations) (Fig. 1a, b; Supplementary Table 1). A scatter plot of the latitudes and elevations of populations shows that northern populations of *C. duclouxiana* tend to occur at elevations and latitudes between those of southern *C. duclouxiana* and *C. gigantea* populations (Supplementary Fig. 1a). Principal component analysis of climate data (obtained from climate stations) further indicates that northern populations of *C. duclouxiana* occur in habitats similar to those of *C. gigantea* that are very different from those of southern populations (Supplementary Fig. 1b), with mean annual precipitation as the major contributor (Supplementary Fig. 1c). Climate records show that both *C. gigantea* and northern *C. duclouxiana* occur in mountain valleys with mean annual precipitation between 640 and 702 mm, and mean annual temperature between 5.85 and 9.1 °C, while southern *C. duclouxiana* occurs in areas with mean annual precipitation between 936 and 1007 mm (with one outlier as 838 mm) and mean annual temperature between 12.6 and 20.8 °C (Supplementary Tables 2, 3).

### Reference transcriptome assembly and DNA polymorphism

The assembled reference transcriptome of *C. duclouxiana* contained 85,401 unigenes with an average length of 676 bp and a contig N50 equaling 1027 bp, and 28,414 of these unigenes were annotated with reference to the NCBI (National Center for Biotechnology Information) nr (non-redundant) database. After aligning transcriptome reads from all individuals to the reference transcriptome and undertaking stringent quality filtering, we identified a total of 878,614 single nucleotide polymorphisms (SNPs) across all 65 individuals of *C. duclouxiana* and *C. gigantea* examined. For 513,615 of these SNPs, covering 16,884 unigenes, there were no missing data across all samples. The transcriptome-wide average values for both indicators of nucleotide diversity, *θ*_π_ and *θ*_w_, were higher for *C. duclouxiana* (0.0031, 0.0151) than for *C. gigantea* (0.0029, 0.0144; Supplementary Table 4).

### Population structure and polymorphism sharing

Phylogenetic analysis of SNP variation showed that samples of *C. gigantea* and *C. duclouxiana* were clustered into separate monophyletic clades (Fig. 1c). Principal component analysis (PCA) based on SNPs also distinguished the two species along PC1 (variance explained = 21.41%, Tracy-Widom *P* = 5.99 × 10^−10^) and further separated northern from southern populations of *C. duclouxiana* along PC2 (variance explained = 4.72%, Tracy-Widom *P* *=* 8.14 × 10^−41^) (Fig. 1d). Analysis of the genetic structure of *C. duclouxiana* and *C. gigantea* using Frappe^45^ assigned most individuals to one or other of two species-specific groups with *K* = 2. However, all individuals representing northern *C. duclouxiana* were of mixed ancestry with a minority of their ancestry derived from *C. gigantea* (Fig. 1e). Some of these individuals were assigned to a third genetic group when *K* = 3 with the remainder indicated to be of mixed ancestry between this third group and the group comprising all southern *C. duclouxiana* individuals (Fig. 1e). When *K* = 4, both *C. gigantea and C. duclouxiana* were each divided into two subgroups with some southern *C. duclouxiana* individuals showing mixed ancestry between northern *C. duclouxiana* and the remainder of southern *C. duclouxiana* (Supplementary Fig. 2).

**Fig. 2:**
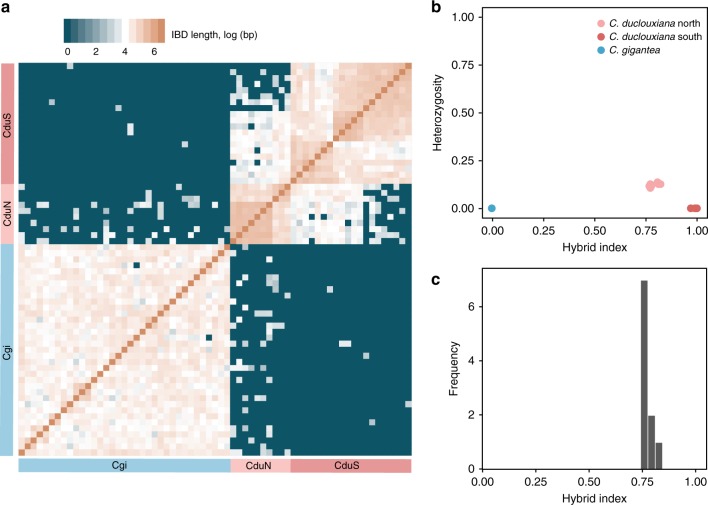
Inference of gene flow between *C. gigantea* and *C. duclouxiana* based on haplotype sharing and hybrid index estimation. **a** Estimated haplotype sharing between individuals of *C. gigantea* (Cgi) and *C. duclouxiana* (CduN: northern population; CduS: southern population). Heatmap colors represent the total length of IBD blocks for each pairwise comparison. **b** Scatter plot of hybrid index (*x*-axis) and heterozygosity (*y*-axis) for 65 individuals of *C. gigantea*, northern *C. duclouxiana* and southern *C. duclouxiana*. Note: there is considerable overlap of dots representing different individuals within each lineage. **c** A histogram showing frequency of hybrid indices for individuals of northern *C. duclouxiana*

The relationships between *C. gigantea* and the northern and southern types of *C. duclouxiana* were further explored by a refined identical-by-descent (IBD) approach^46^. Pairwise comparisons between individuals showed that haplotype sharing was much more common between *C. gigantea* and northern *C. duclouxiana* (50 of 350 pairwise comparisons indicated sharing) than between *C. gigantea* and southern *C. duclouxiana* (only 6 of 700 pairwise comparisons indicated haplotype sharing) (Fig. 2a). As expected, haplotype sharing was most common between northern and southern *C. duclouxiana*, with 143 of 200 pairwise comparisons indicating sharing. In all cases, shared haplotypes were short in length, due to the relatively short length of unigenes in the transcriptome dataset, yet were of sufficient length to reveal intra- and inter-specific gene flow. Finally, a HIest analysis^47^ estimated that northern *C. duclouxiana* individuals had heterozygosity indices between 0.11 and 0.14 (Fig. 2b) and hybrid indices between 0.76 and 0.815 (Fig. 2c), indicating they are advanced generation backcrosses between *C. gigantea* and *C. duclouxiana*. In summary, these results, together with the finding that both *F*_ST_ and *d*_*XY*_ were slightly lower between *C. gigantea* and northern *C. duclouxiana* than between *C. gigantea* and southern *C. duclouxiana* (Supplementary Table 4), strongly indicate that historical gene flow has occurred between *C. gigantea* and *C. duclouxiana* resulting in northern populations of *C. duclouxiana* being composed entirely of advanced generation backcrosses.

### Gene flow and population demography

**Fig. 3:**
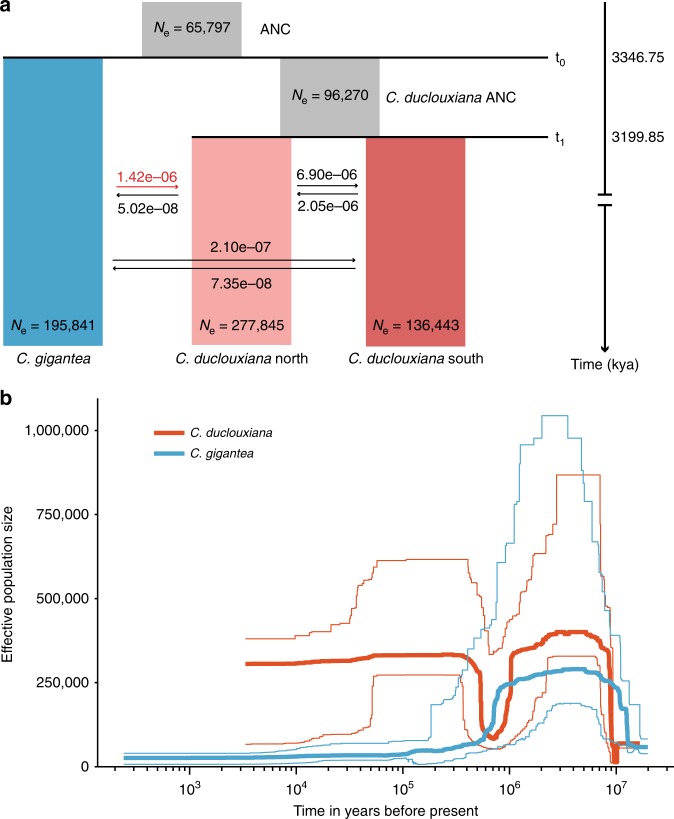
Demographic history of *C. gigantea* and *C. duclouxiana*. **a** Schematic of demographic scenario modeled in *fastsimcoal2*. Estimated effective population sizes (*N*_e_) and divergence times are indicated. The numbers next to arrows denote the per generation migration rate between populations. **b** Changes in effective population size (*N*_e_) over time in *C. gigantea* and *C. duclouxiana* inferred by Stairway Plot method. Thick lines represent the median and thin light lines the 95% pseudo-CI defined by the 2.5% and 97.5% estimations of the SFS analysis

Levels of gene flow among the three genetic groups identified by phylogenetic and population structure analyses were estimated using coalescent-based simulations^48^. Of the 16 candidate migration models compared (Supplementary Fig. 3), the best-fitting one (with maximum Akaike’s weight value, Supplementary Table 5) contained six of the eight possible migration parameters (Fig. 3a). This indicated that *C. gigantea* and *C. duclouxiana* diverged from their common ancestor approximately 3.35 million years ago (Mya), while the northern and southern types of *C. duclouxiana* diverged approximately 3.2 Mya (Fig. 3a; Table 1). Estimated gene flow from *C. gigantea* to the northern type of *C. duclouxiana* was much higher than in the reverse direction or between *C. gigantea* and southern *C. duclouxiana* in either direction, but lower than migration between northern and southern *C. duclouxiana*. The best-fitting model further indicated an absence of historical gene flow between *C. gigantea* and the *C. duclouxiana* ancestral population. A Stairway Plot analysis^49^ showed that population size (*N*_e_) declined in both species until approximately 0.6–1.0 Mya (Fig. 3b). It then expanded rapidly in *C. duclouxiana*, but declined further in *C. gigantea* (Fig. 3b).

**Table 1:** Inferred demographic parameters for the best-fitting demographic model shown in Fig. 3a

Parameters	Point estimation	95% confidence intervals	
		Lower bound	Upper bound
*N*_e-ANC_	65,797	55,016	98,062
*N*_e- *C. duclouxiana* ANC_	96,270	81,860	151,345
*N*_e- *C. gigantea*_	195,841	170,091	207,325
*N*_e-Northern *C. duclouxiana*_	277,845	220,670	302,080
*N*_e-Southern *C. duclouxiana*_	136,443	114,886	173,003
m_*C. gigantea*→northern *C. duclouxiana*_	1.42 × 10^−06^	1.51 × 10^−07^	1.75 × 10^−06^
m_Northern *C. duclouxiana*→*C. gigantea*_	5.02 × 10^−08^	3.14 × 10^−10^	4.91 × 10^−07^
m_*C. gigantea*→southern *C. duclouxiana*_	2.10 × 10^−07^	1.24 × 10^−09^	2.15 × 10^−07^
m_Southern *C. duclouxiana*→*C. gigantea*_	7.35 × 10^−08^	5.25 × 10^−10^	2.57 × 10^−07^
m_Northern *C. duclouxiana*→southern *C. duclouxiana*_	6.90 × 10^−06^	3.44 × 10^−06^	9.60 × 10^−06^
m_Southern *C. duclouxiana*→northern *C. duclouxiana*_	2.05 × 10^−06^	1.11 × 10^−06^	6.57 × 10^−06^
*t*_0_	3,346,750	2,951,850	3,542,350
*t*_1_	3,199,850	2,771,150	3,338,300

*N*_e_: the effective population sizes of each population; *t*: estimated divergence times; *m*: the per generation migration rate between populations

**Fig. 4:**
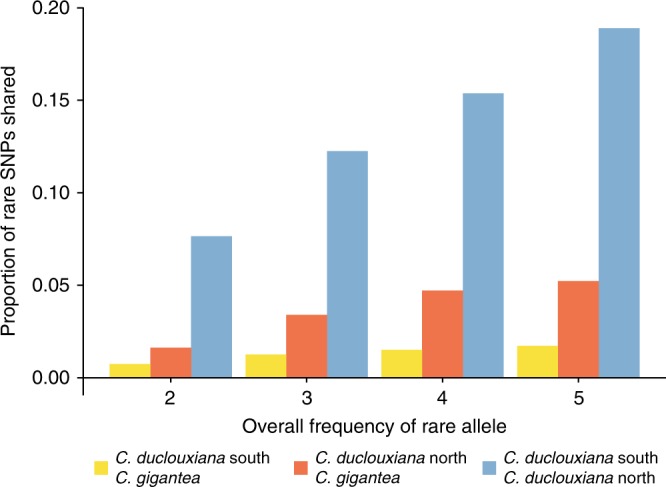
Sharing of rare SNPs among *C. gigantea*, northern *C. duclouxiana*, and southern *C. duclouxiana*. The proportion of all rare SNPs that are only shared between two of the three populations is plotted in each frequency category

Analysis of rare SNPs shared among populations indicated that the proportion of rare alleles shared between northern *C. duclouxiana* and *C. gigantea* was much higher than between southern *C. duclouxiana* and *C. gigantea* (Fig. 4). This suggests that though recent introgression has occurred between *C. gigantea* and both types of *C. duclouxiana* it has been much greater between *C. gigantea* and northern *C. duclouxiana*. The results also indicated that the highest level of recent introgression was between northern and southern *C. duclouxiana* populations (Fig. 4).

### Introgressed loci and positively selected genes

To identify loci introgressed between *C. gigantea* and northern *C. duclouxiana*, we calculated the ABBA-BABA statistic^50,51^ and the modified *f* statistic (*f*_dM_)^52,53^ for all unigenes detected across the whole transcriptome. First, we identified candidate signals of introgression only if the *D*-statistic was greater than or equal to 0.7. Next, we extracted those with averages (over 200 bp windows) of both *D* and *f*_dM_ significantly different from zero (*P* < 0.01). Because introgressed regions generally show lower absolute genetic divergence, we further calculated sequence divergence (*d*_*XY*_) for each candidate introgressed locus and compared this with the whole transcriptome mean *d*_*XY*_. In combination, these tests detected a subset of 1285 candidate loci introgressed from *C. gigantea* into northern *C. duclouxiana*. We performed Gene Ontology (GO) enrichment analysis for these introgressed loci and identified 65 enriched GO terms (corrected *P* < 0.05), including response to salt stress, response to extracellular stimulus, detoxification, and response to metal ion (Supplementary Table 6).

To further explore the genetic basis of possible high elevation adaptation, we performed population branch statistic^54^ and Hudson-Kreitman-Aguadé^55^ tests to identify genes under positive selection within *C. gigantea* and northern *C. duclouxiana*. In total, 730 and 248 positively selected genes were identified within *C. gigantea* and northern *C. duclouxiana*, respectively. GO enrichment analyses identified 46 overrepresented GO terms for positively selected genes within *C. gigantea*, including photosynthesis, light harvesting, regulation of ion transport, and response to ethylene (Supplementary Table 7). For positively selected genes within northern *C. duclouxiana*, 26 GO categories were enriched with some associated with regulation of phosphoprotein phosphatase activity, IMP biosynthetic process, and regulation of proteolysis (Supplementary Table 8).

**Fig. 5:**
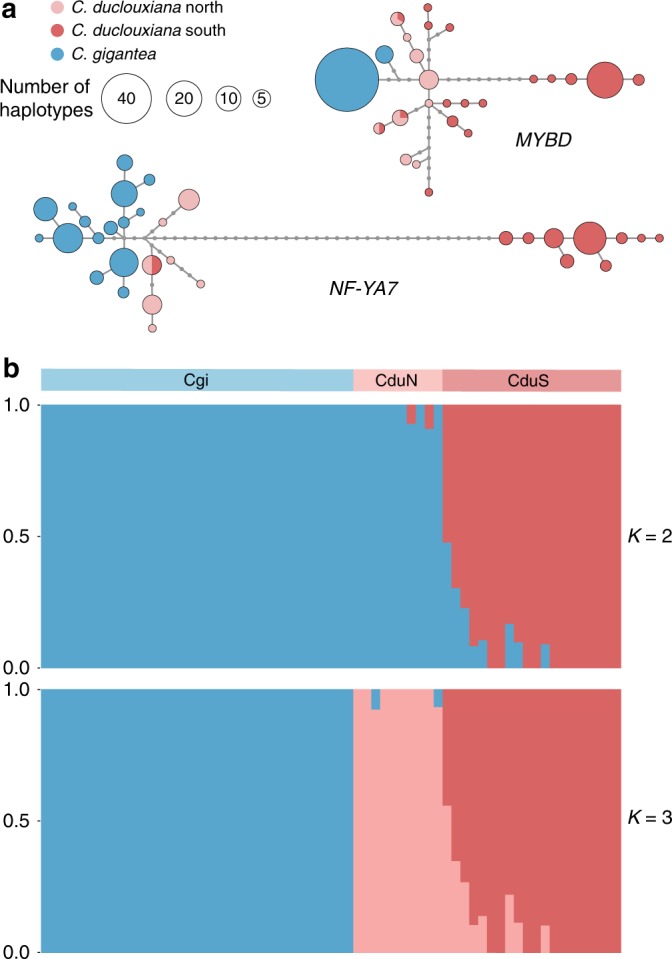
Genetic introgression from *C. gigantea* to *C. duclouxiana*. **a** Haplotype trees for two of 16 introgressed candidate adaptive loci, *MYBD* and *NF-YA7*, generated by Haplotype Viewer (http://www.cibiv.at/∼greg/haploviewer) indicate their introgression from *C. gigantea* into *C. duclouxiana*. **b** Population structure plots with *K* = 2 and *K* = 3 based on SNPs within 16 introgressed candidate adaptive loci (Cgi, *C. gigantea*; CduN, northern *C. duclouxiana*; CduS, southern *C. duclouxiana*)

Sixteen introgressed loci shown to be positively selected, were shared between *C. gigantea* and northern *C. duclouxiana*. To test whether the number of these genes was higher than expected by chance, we randomly sampled 1285, 730, and 248 samples from an available pool of 16,884 samples (without replacement), respectively, and a maximum of 7 overlaps among three samplings were observed after 100,000 replications. These 16 loci were, therefore, considered as introgressed genes likely to be important to the adaptation of northern *C. duclouxiana* to its local environment at high latitudes and elevations on the Qinghai-Tibet Plateau (Fig. 5a; Supplementary Table 9). As a result of the sharing of these 16 positively selected genes between *C. gigantea* and northern *C. duclouxiana*, population structure analyses limited to the 881 SNPs located within these genes (Fig. 5b) revealed a very different picture of population structure to that produced from the analysis of the entire set of 513,615 SNPs (Fig. 1e). Thus, with *K* = 2, northern *C. duclouxiana* individuals formed one genetic group with *C. gigantea*, while all southern *C. duclouxiana* individuals were placed in the second group (Fig. 5b).

### Distribution models for *C. duclouxiana* and *C. gigantea*

**Fig. 6:**
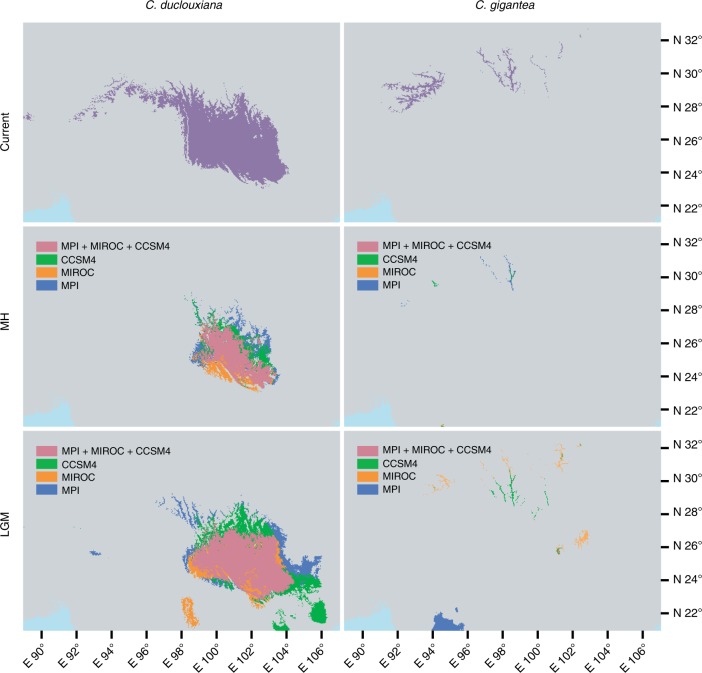
Potential distributions of *C. duclouxiana* and *C. gigantea* predicted by ecological niche modeling for the present, the mid-Holocene and last glacial maximum periods. Ecological niche models for the mid-Holocene and last glacial maximum are based on three general circulation models (GCMs): CCSM4 (green), MIROC (orange), and MPI (blue). The potential distributions supported by all three GCMs are indicated in pink

Jackknife tests of ecological niche models showed that temperature seasonality contributed most to model predictions for the distribution of *C. duclouxiana*, while annual mean temperature and precipitation of driest month contributed most to predicting the distribution of *C. gigantea*. All models exhibited high predictive ability with AUC > 0.9. Based on three general circulation models (GCMs: CCSM4, MIROC, and MPI), the ecological niche models for the middle Holocene and last glacial maximum predicted a wide potential distribution for *C. duclouxiana*, but a narrow one for *C. gigantea*, during both periods (Fig. 6). These different distribution patterns for the two taxa concur with results indicated by the Stairway Plot of effective population sizes (Fig. 3b). For both species, a narrower distribution was predicted for the middle Holocene relative to present-day distributions and that during the last glacial maximum period. Furthermore, the distribution of *C. gigantea* is predicted to have overlapped that of *C. duclouxiana* during the last glacial maximum (Fig. 6). Based on Welch's *t-*test, it is also predicted that the mean elevation for the distribution of *C. duclouxiana* increased gradually with time and is significantly greater now than it was during the middle Holocene and last glacial maximum periods (middle Holocene: *t* = 58.151, d.f. = 15,668, *P* *<* 2.2 × 10^−16^; last glacial maximum: *t* = 69.725, d.f. = 28,776, *P* *<* 2.2 × 10^−16^).

## Discussion

Our large-scale analysis of transcriptome-based SNP variation confirmed that the two cypress species, *Cupressus gigantea* and *C. duclouxiana*, are highly divergent genetically. This was evident from phylogenetic, principal component, and genetic clustering analyses conducted on a large set of transcriptomic SNPs. Our analyses also showed that within *C. duclouxiana* a northern group of populations is genetically divergent from a southern group, and further that individuals of the northern group are of mixed ancestry between *C. gigantea* and southern *C. duclouxiana* with most of their ancestry equating to that of southern *C. duclouxiana*. A similar pattern of differentiation is also evident for morphological characters. Thus, while *C. gigantea* has a wider terminal shoot diameter than both northern and southern types of *C. duclouxiana*, the terminal shoot diameter of northern *C. duclouxiana* is greater than that of southern *C. duclouxiana* (Supplementary Fig. 4). An analysis of demographic history using coalescent-based simulations indicated that *C*. *gigantea* and *C*. *duclouxiana* diverged from their common ancestor approximately 3.35 Mya, while northern and southern types of *C*. *duclouxiana* diverged approximately 3.2 Mya (Fig. 3a; Table 1). An earlier divergence of the two species indicated by a Stairway Plot of changes in effective population sizes over time (Fig. 3b) should be treated with caution, given the reduced accuracy and resolution of this approach for inferring more ancient history^49^. Divergence within this *Cupressus* complex could have been triggered by uplift of southeastern Qinghai-Tibet Plateau, which according to Sun et al.^56^ took place during the late Neogene, although this is disputed by Renner^57^.

Northern *C. duclouxiana* was inferred to be of mixed ancestry in an analysis using Frappe^45^ with *K* = 2 (Fig. 1e), but, rather surprisingly, was shown to be an independent cluster (i.e., not admixed) when *K* = 3. Such a change in genetic pattern has also been observed in some other studies of hybrid lineages. For example, an admixture analysis showed that the Italian sparrow was clearly a hybrid between the House sparrow and the Spanish sparrow when *K* = 2, but indicated it was an independent (non-admixed) genetic group when *K* = 3^58^. Confirmation that individuals of northern *C. duclouxiana* were hybrid emerged from a HIest analysis (Fig. 2b, c) which showed their hybrid indices equated to those expected for advanced generation backcrosses.

Further evidence that northern *C. duclouxiana* (Figs. 1e, 2b) originated from introgression of *C. gigantea* genes into *C. duclouxiana* comes from the greater haplotype sharing between *C. gigantea* and northern *C. duclouxiana*, as revealed by an identity-by-descent (IBD) blocks analysis (Fig. 2a), the slightly lower *F*_ST_ value between *C. gigantea* and northern *C. duclouxiana*, and the marginally higher nucleotide diversity values (*θ*_π_ and *θ*_w_) of northern relative to southern *C. duclouxiana* (Supplementary Table 4). Because it is possible that haplotype sharing might be caused by incomplete lineage sorting from the most recent common ancestor of the two species, rather than by introgression, we conducted an ABBA-BABA analysis, along with tests using a modified *f* statistic and estimates of sequence divergence (*d*_*XY*_) on each unigene, to distinguish between these two possibilities. These analyses clearly identified 1285 loci likely to be introgressed from *C. gigantea* into *C. duclouxiana*.

Additional evidence that introgression has occurred at a much higher rate from *C. gigantea* into northern than southern *C. duclouxiana* came from coalescent-based simulations (Fig. 3a). These indicated that gene flow from *C. gigantea* into northern *C. duclouxiana* (1.42 × 10^−6^) was approximately 7 times greater than into southern *C. duclouxiana* (2.10 × 10^−7^) and that gene flow in the opposite direction was much lower in each case (approximately 28-fold and 20-fold lower, respectively). Highest levels of gene flow were estimated to have occurred, as expected, between northern and southern *C. duclouxiana* (6.90 × 10^−6^ in the forward direction vs. 2.05 × 10^−6^ in the reverse direction).

Of the 1285 loci indicated to have been introgressed from *C. gigantea* into northern *C. duclouxiana*, 16 were positively selected in both *C. gigantea* and northern *C. duclouxiana* but not in southern *C. duclouxiana*. These may be considered as candidate loci that have contributed to the adaptation of *C. gigantea* and northern *C. duclouxiana* to local environments, with some directly involved in the ability of plants to grow at higher latitudes and elevations relative to southern *C. duclouxiana* (Supplementary Table 9). Functional analysis of some of these genes (based on studies conducted on other plants) lends support to this hypothesis. For example, *MYBD* (Fig. 5a) encodes a MYB-like Domain transcription factor, MYBD, that plays a positive role in anthocyanin regulation by stress^59^. Foliar anthocyanins can protect plants facing abiotic or biotic stress, e.g., as sunscreens and antioxidants, thus helping plants to adapt to a wide range of environmental conditions^60^. In *Arabidopsis thaliana*, *MYBD* expression can be stimulated by high light and promote anthocyanin accumulation via inhibiting the expression of *MYBL2*, which encodes a repressor of this process^61^. In addition, *NF-YA7* (Fig. 5a) encodes a multifunctional transcription factor belonging to the NF-YA family which participates in several types of abiotic stress response, including heat, cold, flooding, drought, and nutrient stress^62^. Overexpression of *NF-YA7* in *A. thaliana* leads to the development of a dwarf late-senescent phenotype with enhanced abiotic stress tolerance^62^. Similarly, overexpression of *PtNF*-*YA9* (the *Populus* homolog of *NF-YA7*) produces *A. thaliana* plants that have a small leaf area, a dwarf phenotype and strong tolerance to salt and drought stress^63^. Thus, it is feasible that introgression of particular alleles of these and other genes (Supplementary Table 9) from *C. gigantea* into *C. duclouxiana* enabled *C. duclouxiana* to adapt to cooler and drier conditions at higher latitudes and elevations and extend its range as a consequence. Future studies of the 16 genes identified as candidates of adaptive introgression from *C. gigantea* into northern *C. duclouxiana* should aim to test their adaptive significance in more detail and involve both functional and fitness analyses under common garden and reciprocal transplant conditions.

Although, *C. gigantea* and *C. duclouxiana* are allopatrically distributed in the Qinghai-Tibet Plateau, it is feasible that they have been in contact and exchanged genes at various times since they diverged from each other approximately 3.35 Mya. Our analysis of the demographic history of the two species using *fastsimcoal2* indicated that they did not exchange genes prior to divergence of the northern and southern forms of *C*. *duclouxiana* approximately 3.2 Mya. However, gene flow mainly from *C*. *gigantea* into *C*. *duclouxiana* may have triggered divergence of the two forms of *C. duclouxiana*, and occurred at various times since then.

The effective population sizes of both species have gone through massive changes during the Quaternary most likely as a result of the notable climatic oscillations that occurred during this period. Thus, both species experienced a major bottleneck in effective population size approximately 0.6–1.0 Mya, coinciding with the beginning of the largest glaciation that took place on the Qinghai-Tibet Plateau 0.6–0.8 Mya^64^ (Fig. 3b). However, whereas *C. duclouxiana* largely recovered its former population size following this glaciation, the effective population size of *C. gigantea* has remained relatively unchanged since this event. During this and other glacial periods of the Quaternary, it is feasible that the distributions of the two species were brought together and exchanged genes. Thus, the results of ecological niche modeling indicated that at the time of the last glacial maximum (approximately 0.02 Mya) *C. gigantea* was distributed to the south of its current distribution and overlapped the distribution of *C. duclouxiana* (Fig. 6).

The occurrence of recent gene flow between *C. gigantea* and *C. duclouxiana*, possibly during the last glacial maximum, is supported by the results of an analysis of rare SNP sharing between these two taxa. Because rare alleles are expected, on average, to have originated only recently, they are likely to be restricted to a single population or species in the absence of recent gene flow^65^. In contrast, our analysis revealed that between approximately 2–5% of rare SNPs were shared between *C. gigantea* and northern *C. duclouxiana* while between 1 and 2% were shared between *C. gigantea* and southern *C. duclouxiana*. This indicates that recent gene flow between the species has occurred and was greater between *C. gigantea* and the northern relative to the southern form of *C. duclouxiana*. However, because the geographical distributions of *C. gigantea* and *C. duclouxiana* do not overlap at the current time, and according to our ecological niche model analysis have not done so after the last glacial maximum, introgression is unlikely to have occurred throughout the Holocene.

Our study has revealed a new example of introgression between two conifers in the mountainous region of the eastern Qinghai-Tibet Plateau and adjacent areas. We identified 1285 genes introgressed into northern *C. duclouxiana* from *C. gigantea*. Among these loci, 16 could be related to habitat adaptation, which were positively selected in *C. gigantea* and northern *C. duclouxiana*, but not in southern *C. duclouxiana*. These loci may have contributed, in turn, to the adaptation of northern *C. duclouxiana* to cooler and drier conditions experienced at higher latitudes and elevations. The two cypress species are currently geographically isolated from each other; however, it is likely that they have been in contact and exchanged genes at various times since they diverged from each other approximately 3.35 Mya. Evidence of recent gene flow was obtained from the sharing of rare SNPs between the two species, and may reflect an exchange of genes during the last glacial maximum when the distributions of the two species likely overlapped according to ecological niche models. The results of our study highlight the likely importance of introgression in the adaptation of species to climate change in the past, and emphasize the potential of introgressed populations for adaptation to future climate change.

## Methods

### Sample collection and mRNA sequencing

Leaf samples were collected from a total of 65 individuals comprising 30 *Cupressus duclouxiana* and 35* C. gigantea* trees (Fig. 1a, b; Supplementary Table 1). In addition, one *C. funebris*, one *Juniperus microsperma* and nine *C. chengiana* trees were sampled as outgroups. Young leaves were collected during day-time between 11 a.m. to 3 p.m., frozen instantly using liquid nitrogen and transported to the laboratory. Total RNAs were isolated from each sample using TRIzol reagent (Invitrogen, Carlsbad, CA, USA) and the RNeasy Kit (Qiagen, Hilden, Germany) approach. RNAs with poly (A) tails were purified from total RNA using oligo (dT) magnetic beads before fragmenting into short sequences and synthesizing and purifying cDNAs with a PCR extraction kit (QiaQuick). RNA sequencing was performed using paired-end libraries with 300 bp insert size on an Illumina HiSeq 2000 instrument with 125 bp read length. Raw reads were filtered for downstream analyses, during which reads were removed that contained adapters, poly(N) or were of low-quality.

### Transcriptome de novo assembly and annotation

Filtered reads from one *C. duclouxiana* individual were assembled into contigs using Trinity v2.0.6^66^ with default parameters. Transcripts of less than 200 bp were removed and longest transcripts (in case of alternative splice variants) were selected for the final assembly. CD-HIT-EST v4.6 (https://github.com/weizhongli/cdhit) was used in the final assembly to eliminate redundancies. To obtain high-quality contigs for further annotation and analysis, we removed sequences showing high similarity with known non-coding RNA sequences in the Rfam database (http://rfam.xfam.org/), and also contigs assigned to microbial (MBGD, http://mbgd.genome.ad.jp/), fungal, virus and bacterial sources (sequences downloaded from the NCBI database). In addition, sequences for which 50% of bases aligned with sequences in UTRdb (http://utrdb.ba.itb.cnr.it/) or contained <200 non-UTR bases were excluded. In this way, a final reference set of 85,401 contiguous expressed sequences (unigenes) was obtained. We compared all high-quality unigenes against the NCBI nr database using BLASTX^67^. Functional classification of GO categories for these sequences was performed using the Blast2GO program^68^.

### Reads mapping and SNPs calling

For each *C. duclouxiana* and *C. gigantea* sample, high quality reads were aligned to the reference transcriptome using Bowtie2 v2.2.5 (http://bowtie-bio.sourceforge.net/bowtie2/index.shtml) with default parameters. To identify the ancestral state of these two species and for the identification of positively selected genes (see below), we also mapped the reads of one *C. funebris* sample, one *J. microsperma* sample and nine *C. chengiana* samples to the reference assembly. Picard v1.128 (https://github.com/broadinstitute/picard) was used to remove PCR duplicates and to assign read group information containing library, lane and sample identity. RealignerTargetCreator and IndelRealigner in GATK v3.3 (https://software.broadinstitute.org/gatk/) were used to realign indels. With parameters set as -q 20 -Q 20 -t DP -m 2 -F 0.002, we executed the mpileup command in SAMtools v1.2 (https://github.com/samtools/samtools) to identify SNPs. Sites with coverage depth <2 and >250 and mapping quality <20 were filtered out. SNPs were retained only if they were present in 60 percent of data across all sampled individuals. For all filtered sites in both species, we defined alleles that were the same as those found in *C. funebris* and *J. microsperma* as the ancestral allelic state.

### Phylogenetic and population genetic analysis

A maximum likelihood (ML) phylogenetic tree for *C. duclouxiana* and *C. gigantea* samples, based on SNP variation, was constructed using RAxML v8.1.24^69^ and the GTRGAMMA model for heuristic tree search, with *C. funebris* and *J. microsperma* as outgroups. Estimates of nucleotide diversity, *θ*_π_ (based on pairwise differences between sequences, see ref. ^70^) and *θ*_w_ (based on number of segregating sites between sequences, see ref. ^71^), were calculated using VCFtools v0.1.14 (http://vcftools.sourceforge.net/) and the equations of Watterson^71^, respectively. The pairwise genetic differentiation (*F*_ST_) between species or groups was estimated according to Weir and Cockerham^72^. To further investigate the population structure of *C. duclouxiana* and *C. gigantea*, a principal component analysis (PCA) was conducted on SNP variation using EIGENSOFT v6.0 (https://github.com/DReichLab/EIG) after converting the SNP variant calling format to binary ped format using VCFtools and PLINK v1.07 (http://zzz.bwh.harvard.edu/plink/). Significance levels of principal components were determined using the Tracy-Widom test and the first two significant components were plotted. In addition, the software Frappe v1.1^45^ was used to examine population structure with number of clusters (*K*) ranging from 2 to 10 and each run comprising 10,000 iterations. Also, a refined identity-by-descent (IBD) blocks analysis^46^ was performed using the algorithm from BEAGLE v4.1 (window = 600 overlap = 50 ibd = true ibdtrim = 20 ibdlod = 3.0) to detect shared haplotypes between individuals of both species. Finally, the heterozygosity and hybrid index of each of the 65 individuals were calculated using the R package HIest^47^ on 144,027 SNPs that had no missing data for each parental population (*C. gigantea* and southern *C. duclouxiana*, respectively) and for which parental populations had allele frequency differences exceeding 0.4.

### Demographic history

We used *fastsimcoal2*^48^ to infer divergence times and gene flow based on the multidimensional site frequency spectrum for *C. gigantea* and the northern and southern populations of *C. duclouxiana*. To minimize the effects of selection on demographic inference, only SNPs at fourfold degenerate sites (with no missing data across all individuals sampled) were analyzed. SNPs located within 5 bp of each other were also excluded, as were those that significantly deviated (*P* < 0.05) from Hardy-Weinberg equilibrium when tested using VCFtools. The mutation rate per site per generation was estimated as: *µ* = *D* × *g*/2*T*, where *D* is the observed frequency of pairwise differences between the two species, *T* is the estimated divergence time and *g* is the estimated generation time. Average generation time (*g*) was set to 50 years and estimated divergence time between *Juniperus* and *Cupressus* was set to 62.8 Mya according to previous field surveys and studies of other Cupressaceae species^73^, respectively. These values yielded an estimated mutation rate of 7.0 × 10^−9^ mutations per site per generation. Parameter estimates were obtained using the composite ML approach for 16 models that differed in presence or absence of migration (Supplementary Fig. 3). Global ML estimates were derived from 40 independent runs, with 50,000–100,000 coalescent simulations and 10–40 likelihood maximization algorithm cycles. Akaike’s information criterion and Akaike’s weight of evidence were used to assess the relative fit of each model^48^. We chose the model with the highest Akaike’s weight as the one of best fit and constructed 90% nonparametric bootstrap confidence intervals by sampling the fourfold degenerate SNP matrix with replacement. In addition, a Stairway Plot analysis was performed to investigate changes in effective population size over time for both *C. gigantea* and *C. duclouxiana*, using fourfold degenerate SNP frequency spectra for each species, respectively^49^. Two hundred SFS subsamples were generated with each containing a random selection of 2/3 sites and the median of these was used as the final estimation.

To test the relative levels of gene flow between *C. gigantea* and northern and southern *C. duclouxiana*, the proportion of rare SNPs shared among the three types was calculated. We defined SNPs with frequencies from 2 (1.54%) to 5 (3.85%) in all 65 individuals, i.e., 130 alleles, as rare and calculated the proportion of them shared only between two of the three populations in each frequency category. Because rare alleles are expected on average to have originated only recently, they will be limited to a single population or species if recent gene flow is absent^65^. Hence, this approach helps to determine whether introgression has occurred recently between the species and populations examined.

### Screening for introgressed genes

Based on the tree topology presented in Supplementary Fig. 5, we employed both Patterson’s *D* statistic^50,51^ and a modified *f* statistic (*f*_dM_)^52,53^ to identify potential loci introgressed from *C. gigantea* to the northern population of *C. duclouxiana*, using *C. funebris* and *J. microsperma* as outgroups. The *D* statistic was used to examine the phylogenetic distribution of derived alleles at loci that display either an ABBA or BABA allelic configuration using Eq. (1):

1 $$D(P_1,P_2,P_3,O) = \frac{{\mathop {\sum }\nolimits_{i = 1}^n \left( {\left( {1 - \hat p_{i1}} \right)\hat p_{i2}\hat p_{i3}\left( {1 - \hat p_{i4}} \right) - \hat p_{i1}\left( {1 - \hat p_{i2}} \right)\hat p_{i3}(1 - \hat p_{i4})} \right)}}{{\mathop {\sum }\nolimits_{i = 1}^n \left( {\left( {1 - \hat p_{i1}} \right)\hat p_{i2}\hat p_{i3}\left( {1 - \hat p_{i4}} \right) + \hat p_{i1}\left( {1 - \hat p_{i2}} \right)\hat p_{i3}(1 - \hat p_{i4})} \right)}},$$

where *P*_1_, *P*_2_, *P*_3_ and *P*_4_ (O) are the four taxa compared and *p*_*ij*_ is the derived allele frequency of a site *i* in population *j*.

The modified *f* statistic, *f*_dM_, was calculated based on Eq. (2):

2 $$S\left( {P_1;P_2;P_3;O} \right) = \mathop {\sum }\limits_i (\left( {1 - p_{i1}} \right)p_{i2}p_{i3}\left( {1 - p_{i4}} \right)) - \mathop {\sum }\limits_i (p_{i1}\left( {1 - p_{i2}} \right)p_{i3}(1 - p_{i4}))$$

If *p*_*i*2_ > = _*pi*1_, then *f*_dM_ = *S*(*P*_1_;*P*_2_;*P*_3_;O)/*S*(*P*_1_;*P*_D_;*P*_D_;O) and *P*_D_ is the population (*P*_2_ or *P*_3_) that has the higher frequency of the derived allele; if *p*_*i*2_ < *p*_*i*1_, then *f*_dM_ = *S*(*P*_1_;*P*_2_;*P*_3_;O)/-*S*(*P*_D_;*P*_2_;*P*_D_;O) and *P*_D_ is the population (*P*_1_ or *P*_3_) that has the higher frequency of the derived allele^53^.

For each of all the assembled unigenes, the standard error was calculated using a moving block bootstrap approach with optimal block size being 200 bp. All tests were followed by two tailed *Z*-tests to determine if each *D* or *f*_dM_ value was significantly different from zero, indicating potential gene flow.

To rule out false positive introgressed loci, resulting from incomplete lineage sorting, mean pairwise sequence divergence (*d*_*XY*_) of the whole transcriptome and *d*_*XY*_ for each unigene were calculated between *C. gigantea* and northern *C. duclouxiana* as a complementary analysis to the *D* statistic and modified *f* statistic^74^, using python scripts (https://github.com/simonhmartin/genomics_general; see ref. ^52^). For each candidate introgressed locus, *d*_*XY*_ was calculated using a smaller block size of 200 bp for moving block bootstrapping. If the mean *d*_*XY*_ of a putatively introgressed locus was lower than the mean value of the whole transcriptome, the two values were compared statistically using a Mann–Whitney *U*-test. The Benjamini-Hochberg false discovery rate method was performed to decrease false positive rate for all tests (*D* statistic, *f* statistic and *d*_*XY*_; *P* *<* 0.01).

Having identified candidate introgressed loci in the above way, GO enrichment analysis was conducted using the TopGO package in Bioconductor (http://www.bioconductor.org). Fisher’s exact tests with weight01 algorithms were employed to examine the significance of enrichment, with corrected *P*-values < 0.05 considered significant.

### Identification of positively selected genes

Two methods, the population branch statistic^54^ and the Hudson-Kreitman-Aguadé test^55^, were applied to identify genes under positive selection within two target populations, *C. gigantea* and northern *C. duclouxiana*, separately. Samples from southern *C. duclouxiana* were assumed as the control population. As two populations (MSZ-43 and MSZ-51) from southern *C. duclouxiana* may have been recipients of gene flow from northern *C. duclouxiana* (Supplementary Fig. 2), we discarded them within these analyses to avoid spurious signals of positive selection. We carried out the population branch statistics for two triples, *C. gigantea*-southern *C. duclouxiana*-outgroup and northern *C. duclouxiana*-southern *C. duclouxiana*-outgroup. Nine individuals of *C. chengiana* are assumed as an outgroup population. For each unigene, we calculated *F*_ST_ between population pairs including the target population and control population, the target population and outgroup and the control population and outgroup. The population branch statistic (PBS) value of the target population was calculated using Eq. (3):

3 $${\mathrm{PBS}}_{{\mathrm{pop}}T} = \frac{{T^{{\mathrm{pop}}T - C} + T^{{\mathrm{pop}}T - O} - T^{{\mathrm{popC}} - O}}}{2},$$

where *T* = −log(1–*F*_ST_) is the population divergence time *T* in units scaled by the population size.

For the Hudson-Kreitman-Aguadé tests, the number of polymorphic sites in the target population (*C. gigantea* or northern *C. duclouxiana*) was denoted as A and the number of fixed differences between the target population and both control populations (southern *C. duclouxiana*) and outgroup (*C. funebris*) was denoted as B. We compared the ratio of A/B for each unigene to the transcriptome-wide average and tested the null hypothesis A(unigene)/B(unigene) = A(transcriptome-wide)/B(transcriptome-wide) using Pearson's chi-square test for the 2 × 2 contingency table.

Unigenes with the highest 10% of the population branch statistic and a significant *P*-value (<0.05) for the Hudson-Kreitman-Aguadé test were recognized as positively selected genes in *C. gigantea*/northern *C. duclouxiana*. We carried out the GO enrichment analysis for positively selected genes using the same method applied to candidate introgressed loci.

### Morphological differentiation and habitat differentiation

To quantify morphological differentiation between *C. gigantea*, northern and southern *C. duclouxiana*, we compared the diameter of the last ramification shoots with leaves. Nine individuals across three populations for each group were examined, and for each individual five of the last ramification shoots were randomly collected, preserved as herbarium specimens and measured. Mann–Whitney *U*-test was used to test the differences of diameters among groups.

To better illustrate the habitat differentiation among the three groups, a scatter plot for latitude against elevation of all populations was constructed. Subsequently, we conducted a principal component analysis on climate data (26 climate variables) obtained from ten climate stations (Supplementary Tables 2, 3) located within the sampling areas of the three groups, from the National Meteorological Information Center, China (http://data.cma.cn). Principal component analysis and graphical illustrations were performed with R v3.4.0.

### Ecological niche modeling

To explore the distributional shifts of the two *Cupressus* species in response to recent Quaternary climate change, we conducted ecological niche modeling with MAXENT v3.3.4^75^. This predicted the potential distribution of *C. duclouxiana* and *C. gigantea* at present, during the mid-Holocene (0.006 Mya) and at the last glacial maximum (0.022 Mya) using the following parameters: random test points = 25; replicates = 20; type = subsample; maximum iterations = 5000. Information from 56 presence sites for *C. duclouxiana* and 17 for *C. gigantea* were collected from field investigations and herbarium records (http://www.gbif.org). Data for 19 bioclimatic variables for each period were retrieved from the WorldClim database (http://www.worldclim.com) with a resolution of 2.5 arc-min. For the mid-Holocene and last glacial maximum, paleoclimate data, based on three different GCMs (CCSM4, MIROCESM, MPI-ESM-P), were employed. To avoid multicollinearity, we examined pairwise Pearson correlations (*r*) among the bioclimatic variables and eliminated one of the variables in each pair with *r* > 0.7. Seven variables were finally retained (Bio1 annual mean temperature, Bio2 mean diurnal range, Bio3 isothermality, Bio4 temperature seasonality, Bio13 precipitation of wettest month, Bio14 precipitation of driest month, Bio15 precipitation seasonality). The AUC (area under the receiver operating characteristic curve) values were used to compare predictive performance of one model with another and values above 0.9 indicated better model performance. Jackknife tests were also performed to identify which variables contributed the most individually. In addition, we downloaded altitude information from the SRTM elevation database (https://www2.jpl.nasa.gov/srtm/). The elevation for each predicted species distribution model (SDM) was extracted using QGIS v2.18.10 (https://qgis.org/en/site/). Welch’s *t*-test was employed to examine divergence between the average altitude values of the SDMs for *C. duclouxiana*.

Changes in distributional shifts of the two species in response to recent Quaternary climate change as revealed by our ecological modeling analysis provide an environmental context to the effects of adaptive introgression on the recent demography of these two species.

### Statistics and reproducibility

Pearson's chi-square test and Welch’s *t*-test were performed using R v3.4.0. *Z*-test was performed using perl module Statistics::Zed. Mann-Whitney *U*-test was performed using perl module Statistics::Test::WilcoxonRankSum. Benjamini-Hochberg FDR correction was performed using perl module Statistics::Multtest. All the results within this study are reproducible.

### Reporting summary

Further information on research design is available in the Nature Research Reporting Summary linked to this article.

## Supplementary information

Supplementary Information

Reporting Summary

## Data Availability

The transcriptome sequencing data have been deposited in the Short Read Archive at NCBI under the accession codes SAMN08634857 to SAMN08634891, SAMN08634904 to SAMN08634933, SAMN08638057, and SAMN08638058.

## Code availability

The custom scripts have been made available at https://github.com/mayz11/cypress.
