# Supplementary material for: Ancient introgression drives adaptation to cooler and drier mountain habitats in a cypress species complex
Source: Commun Biol. 2019 Jun 18;2:213. doi: 10.1038/s42003-019-0445-z (PMC6581913; doi:10.1038/s42003-019-0445-z)
Supplement: Supplementary file 1 — Supplementary Information [file 42003_2019_445_MOESM1_ESM.pdf]

Supplementary Figures

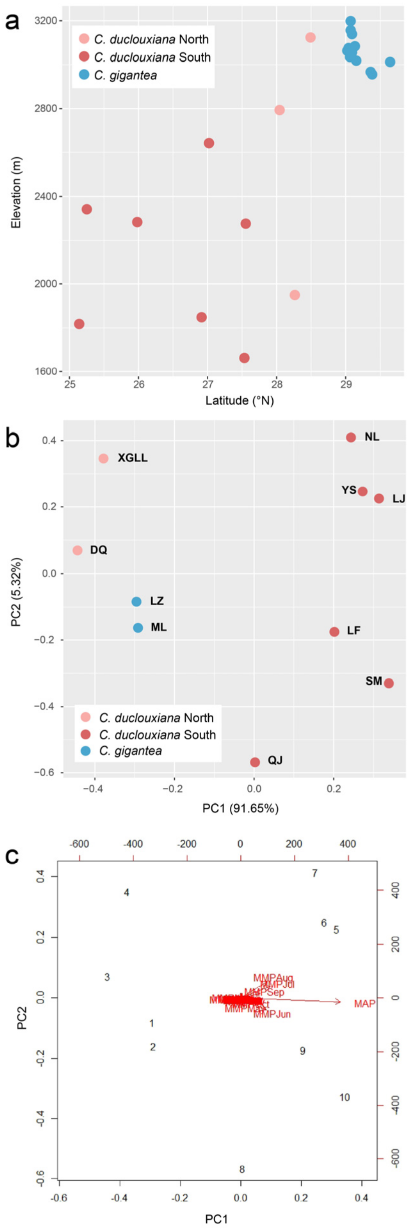

Supplementary Figure 1 Habitat differentiation of *Cupressus gigantea*, northern and southern *C. duclouxiana*. (a) A scatter plot for the latitude (*x*-axis) and elevation (*y*-axis) of the three groups, (b) principal component analysis (PCA) plots of the first two components of climate data from climate stations at or close to the sampling sites of the three groups, and (c) the contributions of 26 factors of climate data (mean annual precipitation (MAP), mean monthly precipitation (MMP) of each month, mean annual temperature (MAT), mean monthly temperature (MMT) of each month) to the two major principal components in the PCA plots as shown in (b). Note that in (b), each dot represents a climate station. Abbreviations: LZ, Linzhi, Xizang Autonomous Region; ML: Milin, Xizang Autonomous Region; DQ, Deqing, Yunnan; XGLL, Xianggelila, Yunnan; LJ, Lijiang, Yunnan; YS, Yongsheng, Yunnan; NL, Ninglang, Yunnan; QJ, Qiaojia, Yunnan; LF, Lufeng, Yunnan; SM, Songming, Yunnan. The source data were listed in Supplementary Tables 2 and 3.

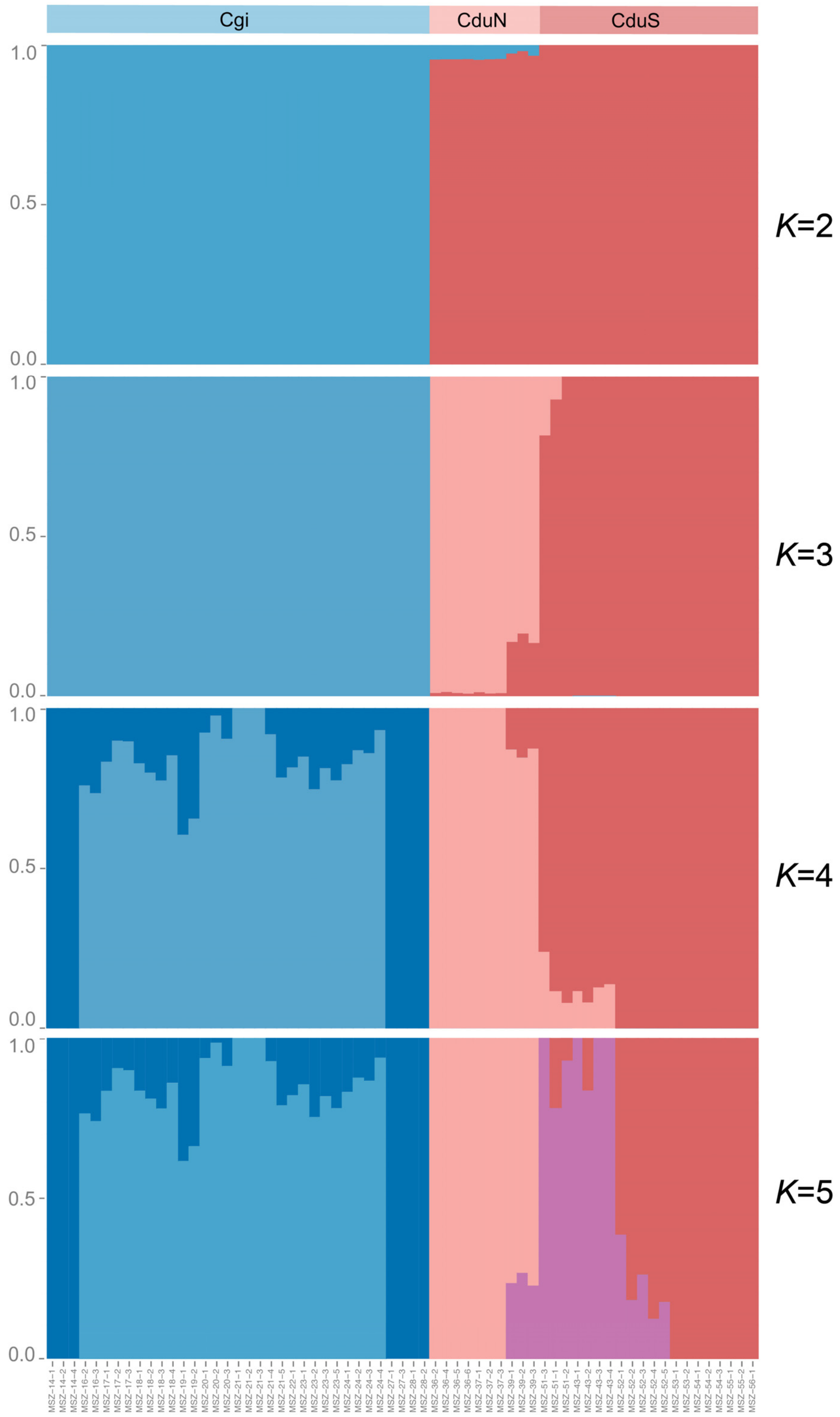

Supplementary Figure 2 Population structure plots from *K*=2 to *K*=5. The ID of samples from *C. gigantea* (Cgi), northern *C. duclouxiana* (CduN) and southern *C. duclouxiana* (CduS) are presented at the bottom of the plots.

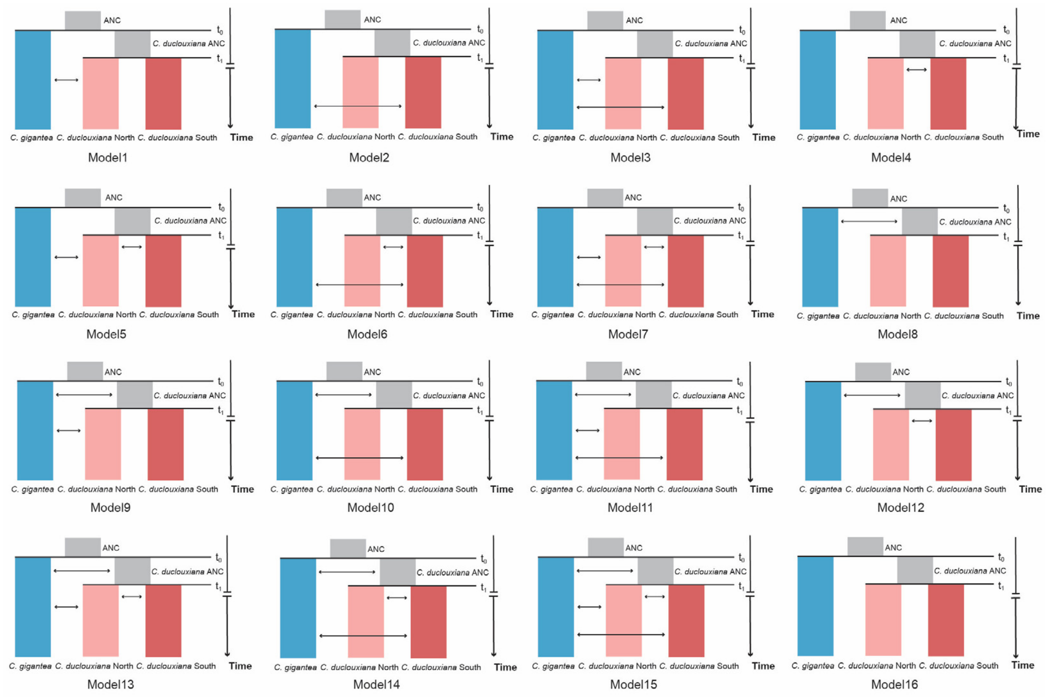

Supplementary Figure 3 Schematic diagram of tested demographic models using *fastsimcoal2*.

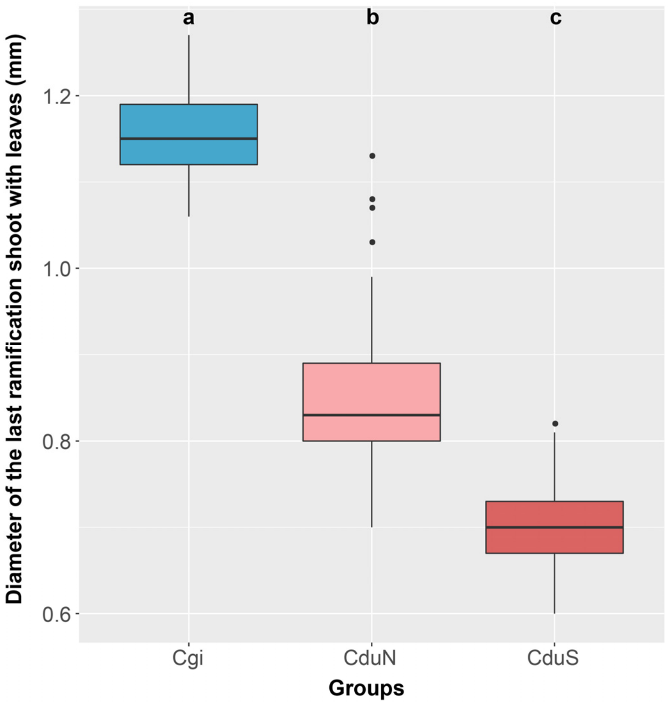

Supplementary Figure 4 Box plots of variations of the diameter of the last ramification shoot with leaves for northern (CduN) and southern *C. duclouxiana* (CduS) and *C. gigantea* (Cgi). The boxes indicate 25th and 75th percentiles, and the lines in the middle of the box indicates the median values. Different letters on the top of the figure indicate significant differences among groups (P < 0.001, Mann-Whitney U-test).

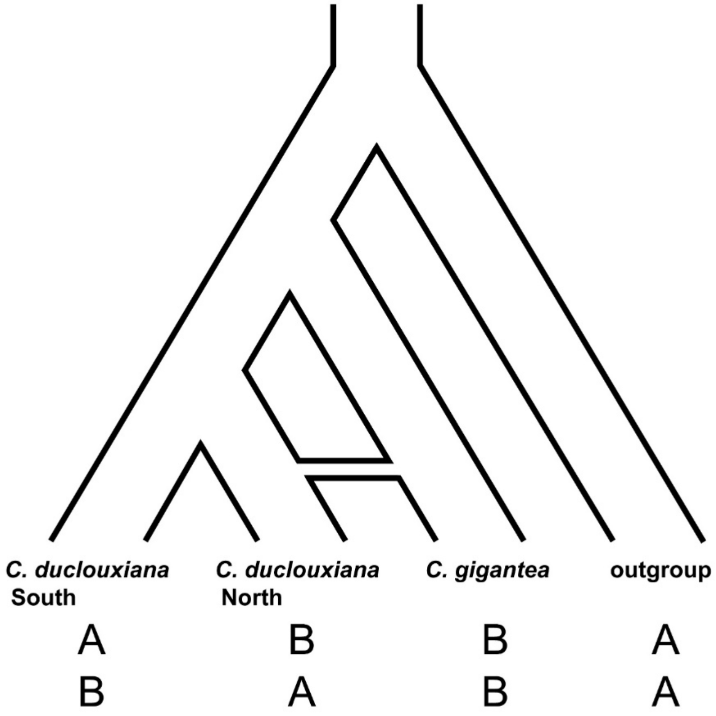

Supplementary Figure 5 Tree model used in ABBA-BABA analysis.

Supplementary Tables

Supplementary Table 1 Overview of sample information.

Population ID	Sample ID	Species	Latitude	Longitude	Elevation
1	MSZ-14-1	*C. gigantea*	N29.62°	E94.40°	3004.46m
1	MSZ-14-2	*C. gigantea*	N29.62°	E94.40°	3004.46m
1	MSZ-14-4	*C. gigantea*	N29.62°	E94.40°	3004.46m
2	MSZ-16-2	*C. gigantea*	N29.13°	E93.85°	3010.71m
2	MSZ-16-3	*C. gigantea*	N29.13°	E93.85°	3010.71m
3	MSZ-17-1	*C. gigantea*	N29.07°	E93.39°	3047.48m
3	MSZ-17-2	*C. gigantea*	N29.07°	E93.39°	3047.48m
3	MSZ-17-3	*C. gigantea*	N29.07°	E93.39°	3047.48m
4	MSZ-18-1	*C. gigantea*	N29.04°	E93.34°	3027.05m
4	MSZ-18-2	*C. gigantea*	N29.04°	E93.34°	3027.05m
4	MSZ-18-3	*C. gigantea*	N29.04°	E93.34°	3027.05m
4	MSZ-18-4	*C. gigantea*	N29.04°	E93.34°	3027.05m
5	MSZ-19-1	*C. gigantea*	N29.02°	E93.32°	3067.43m
5	MSZ-19-2	*C. gigantea*	N29.02°	E93.32°	3067.43m
6	MSZ-20-1	*C. gigantea*	N29.11°	E93.15°	3076.08m
6	MSZ-20-2	*C. gigantea*	N29.11°	E93.15°	3076.08m
6	MSZ-20-3	*C. gigantea*	N29.11°	E93.15°	3076.08m
7	MSZ-21-1	*C. gigantea*	N29.05°	E93.07°	3148.90m
7	MSZ-21-2	*C. gigantea*	N29.05°	E93.07°	3148.90m
7	MSZ-21-3	*C. gigantea*	N29.05°	E93.07°	3148.90m
7	MSZ-21-4	*C. gigantea*	N29.05°	E93.07°	3148.90m
7	MSZ-21-5	*C. gigantea*	N29.05°	E93.07°	3148.90m
8	MSZ-22-1	*C. gigantea*	N29.07°	E92.93°	3131.12m
9	MSZ-23-1	*C. gigantea*	N29.05°	E93.16°	3190.48m
9	MSZ-23-2	*C. gigantea*	N29.05°	E93.16°	3190.48m
9	MSZ-23-3	*C. gigantea*	N29.05°	E93.16°	3190.48m
9	MSZ-23-5	*C. gigantea*	N29.05°	E93.16°	3190.48m
10	MSZ-24-1	*C. gigantea*	N29.00°	E93.23°	3055.41m
10	MSZ-24-2	*C. gigantea*	N29.00°	E93.23°	3055.41m
10	MSZ-24-3	*C. gigantea*	N29.00°	E93.23°	3055.41m
10	MSZ-24-4	*C. gigantea*	N29.00°	E93.23°	3055.41m
11	MSZ-27-1	*C. gigantea*	N29.34°	E94.38°	2959.04m
11	MSZ-27-3	*C. gigantea*	N29.34°	E94.38°	2959.04m
12	MSZ-28-1	*C. gigantea*	N29.36°	E94.40°	2948.23m
12	MSZ-28-2	*C. gigantea*	N29.36°	E94.40°	2948.23m
13	MSZ-36-2	*C. duclouxiana*	N28.49°	E98.82°	2771.59m
13	MSZ-36-4	*C. duclouxiana*	N28.48°	E98.84°	2912.90m

13	MSZ-36-5	*C. duclouxiana*	N28.48°	E98.84°	2944.62m
13	MSZ-36-6	*C. duclouxiana*	N28.47°	E98.85°	3116.22m
14	MSZ-37-1	*C. duclouxiana*	N28.24°	E98.86°	1941.01m
14	MSZ-37-2	*C. duclouxiana*	N28.24°	E98.86°	1941.01m
14	MSZ-37-3	*C. duclouxiana*	N28.24°	E98.86°	1941.01m
15	MSZ-39-1	*C. duclouxiana*	N28.02°	E99.44°	2785.76m
15	MSZ-39-2	*C. duclouxiana*	N28.02°	E99.44°	2785.76m
15	MSZ-39-3	*C. duclouxiana*	N28.02°	E99.44°	2785.76m
16	MSZ-43-1	*C. duclouxiana*	N26.89°	E99.96°	1840.56m
16	MSZ-43-2	*C. duclouxiana*	N26.89°	E99.96°	1840.56m
17	MSZ-43-3	*C. duclouxiana*	N26.93°	E99.96°	1834.29m
17	MSZ-43-4	*C. duclouxiana*	N26.93°	E99.96°	1834.29m
18	MSZ-51-1	*C. duclouxiana*	N27.00°	E100.23°	2633.40m
18	MSZ-51-2	*C. duclouxiana*	N27.00°	E100.23°	2633.40m
18	MSZ-51-3	*C. duclouxiana*	N27.00°	E100.23°	2633.40m
19	MSZ-52-1	*C. duclouxiana*	N25.96°	E100.39°	2275.79m
19	MSZ-52-2	*C. duclouxiana*	N25.96°	E100.39°	2275.79m
19	MSZ-52-3	*C. duclouxiana*	N25.96°	E100.39°	2275.79m
19	MSZ-52-4	*C. duclouxiana*	N25.96°	E100.39°	2275.79m
19	MSZ-52-5	*C. duclouxiana*	N25.96°	E100.39°	2275.79m
20	MSZ-53-1	*C. duclouxiana*	N25.12°	E101.80°	1810.52m
20	MSZ-53-2	*C. duclouxiana*	N25.12°	E101.80°	1810.52m
21	MSZ-54-1	*C. duclouxiana*	N25.23°	E102.74°	2333.95m
21	MSZ-54-2	*C. duclouxiana*	N25.23°	E102.74°	2333.95m
21	MSZ-54-3	*C. duclouxiana*	N25.23°	E102.74°	2333.95m
22	MSZ-55-1	*C. duclouxiana*	N27.53°	E102.31°	2268.34m
22	MSZ-55-2	*C. duclouxiana*	N27.53°	E102.31°	2268.34m
23	MSZ-56-1	*C. duclouxiana*	N27.51°	E102.23°	1654.54m

Supplementary Table 2 Mean annual precipitation and mean monthly precipitation for each month averaged over 30 years (1981-2010) that were collected from climate stations in the sampling areas of *C. gigantea* (Cgi), and northern (CduN) and southern (CduS) populations of *C. duclouxiana*. The climate data were downloaded from National Meteorological Information Center, China (http://data.cma.cn). Abbreviations: MAP, mean annual precipitation; MMP, mean monthly precipitation; LZ, Linzhi, Xizang Autonomous Region; ML: Milin, Xizang Autonomous Region; DQ, Deqing, Yunnan; XGLL, Xianggelila, Yunnan; LJ, Lijiang, Yunnan; YS, Yongsheng, Yunnan; NL, Ninglang, Yunnan; QJ, Qiaojia, Yunnan; LF, Lufeng, Yunnan; SM, Songming, Yunnan. Units for MAP and MMP, mm.

Group	Station code	MAP	MMP Jan.	MMP Feb.	MMP Mar.	MMP Apr.	MMP May	MMP June	MMP July	MMP Aug.	MMP Sept.	MMP Oct.	MMP Nov.	MMP Dec.
Cgi	LZ	692.5	1.3	4.4	19	46.2	75.3	119.2	143.3	122.2	110.5	45.4	4.7	1
Cgi	ML	702.2	3.3	12.3	28.2	55.7	81.2	117.4	148.4	113	88.9	44.8	5.8	3.2
CduN	DQ	640	7.8	19.2	51.6	64.2	55.7	59.5	126	111.7	68.5	48.5	21.2	6.1
CduN	XGLL	650.8	10.1	16.5	33.6	30.9	37	76.8	154.4	146.7	83.7	45.1	11.3	4.7
CduS	LJ	980.2	4.4	4.2	9.4	18.3	55.7	164.1	241.8	213.5	150.4	58.1	13	3.2
CduS	YS	950.8	4	5.7	14.6	18.2	66	165.1	242.3	215.7	165.5	66.3	13.4	3.4
CduS	NL	936.1	5.7	5	10.1	14.8	54.9	139.9	252.4	220	155.7	74.3	15.2	2.8
CduS	QJ	838.2	12.8	10	17.5	23.1	74.1	179.9	167.1	112.3	126.2	87.3	20.3	7.6
CduS	LF	937.2	13.5	15.3	15.4	25.6	83.7	159	198.9	184.7	118.8	77.7	33.7	10.9
CduS	SM	1006.9	16.6	15.3	19	25.6	90.4	189.1	212.4	185.5	122.6	80.2	37	13.2

Supplementary Table 3 Mean annual temperature and mean monthly temperature for each month averaged over 30 years (1981-2010) that were collected from climate stations in the sampling areas of *C. gigantea* (Cgi), and northern (CduN) and southern (CduS) populations of *C. duclouxiana*. The climate data were downloaded from National Meteorological Information Center, China (http://data.cma.cn). Abbreviations: MAT, mean annual temperature; MMT, mean monthly temperature; LZ, Linzhi, Xizang Autonomous Region; ML: Milin, Xizang Autonomous Region; DQ, Deqing, Yunnan; XGLL, Xianggelila, Yunnan; LJ, Lijiang, Yunnan; YS, Yongsheng, Yunnan; NL, Ninglang, Yunnan; QJ, Qiaojia, Yunnan; LF, Lufeng, Yunnan; SM, Songming, Yunnan. Units for MAT and MMT, ˚C.

Group	Station code	MAT	MMT Jan.	MMT Feb.	MMT Mar.	MMT Apr.	MMT May	MMT June	MMT July	MMT Aug.	MMT Sept.	MMT Oct.	MMT Nov.	MMT Dec.
Cgi	LZ	9.1	1	2.8	5.9	8.9	12.1	15.1	16.2	15.7	13.9	10.3	5.5	1.7
Cgi	ML	8.6	0.2	2.1	5.3	8.2	11.5	14.8	16	15.7	13.8	9.8	4.8	1
CduN	DQ	5.85	-1.8	-0.9	1.55	4.6	9.1	12.45	13	12.65	10.95	6.95	2.4	-0.65
CduN	XGLL	6.3	-2.3	-0.4	2.6	5.8	9.9	13.5	13.9	13.3	11.7	7.4	2	-1.6
CduS	LJ	12.9	4.1	6.5	9.9	13.3	17.2	19.4	19.1	18.5	16.7	13.4	8.3	4.5
CduS	YS	13.7	6.4	8	10.7	13.6	16.6	18.6	18.2	17.5	15.9	13.6	9.5	6.6
CduS	NL	12.6	6.4	8.7	11.7	14.9	18.3	19.5	19.1	18.5	16.9	14.5	9.8	6.3
CduS	QJ	20.8	12.4	15.6	20.3	24.4	25.7	25.9	26.4	26.2	23.1	19.8	16.7	12.7
CduS	LF	16.2	8.5	10.6	14	17.8	20.7	21.9	21.6	21	19.7	17.2	12.6	8.9
CduS	SM	14.4	7.3	9.1	12.5	16.1	18.5	19.6	19.6	19.2	17.4	14.9	10.8	7.5

Supplementary Table 4 Population genetic summary statistics.

Population	θ_π	θ_w	F_{ST}		d_{XY}	
			C. duclouxiana north	*C. gigantea*	*C. duclouxiana* north	*C. gigantea*
C. gigantea	0.0029	0.0144	-	-	-	-
C. duclouxiana	0.0031	0.0151	-	0.3054	-	0.0122
C. duclouxiana north population	0.0034	0.0192	-	0.3060	-	0.0119
C. duclouxiana south population	0.0029	0.0162	0.1399	0.3126	0.0078	0.0124

Supplementary Table 5 Relative likelihood of the candidate models shown in Supplementary Figure 3. [a] Based on the best likelihood among the 40 independent runs for each model. [b] The calculation of AIC_i , Δ_i and w_i are according to the methods shown in ref[1].

Model ID	$Max(log_{10}(Lhood_i))$[a]	No. of parameters (d)	AIC_i[b]	Δ_i[b]	Model normalized relative likelihood (w_i)[b]
Model1	-108567	9	499986.6	2702.792	0
Model2	-108614	9	500205.2	2921.372	0
Model3	-108572	11	500014.1	2730.32	0
Model4	-108029	9	497509.7	225.887	8.88E-50
Model5	-108000	11	497380.4	96.632	1.04E-21
Model6	-108065	11	497677.8	393.933	2.87E-86
Model7	-107978	13	497283.8	0	0.998716363
Model8	-108588	9	500085.7	2801.863	0
Model9	-108432	11	499370.4	2086.581	0
Model10	-108584	11	500070.1	2786.273	0
Model11	-108536	13	499851.7	2567.894	0
Model12	-108043	11	497577.9	294.116	1.36E-64
Model13	-107981	13	497297.1	13.314	0.001283637
Model14	-108037	13	497552.9	269.057	3.75E-59
Model15	-108003	15	497404.4	120.543	6.67E-27
Model16	-108573	7	500009	2725.193	0

Supplementary Table 6 GO enrichment of introgressed genes in *C. duclouxiana* northern population.

Class	GO ID	Term	Annotated	Introgressed genes	Corrected *P*-value
BP	GO:0048519	negative regulation of biological process	239	17	0.00038
BP	GO:0006486	protein glycosylation	63	10	0.00044
BP	GO:0008033	tRNA processing	58	9	0.00051
BP	GO:0043628	ncRNA 3'-end processing	23	5	0.00094
BP	GO:0006753	nucleoside phosphate metabolic process	228	8	0.00221
BP	GO:0043043	peptide biosynthetic process	653	19	0.0023
BP	GO:2000280	regulation of root development	13	4	0.0024
BP	GO:0007186	G-protein coupled receptor signaling pathway	15	4	0.00425
BP	GO:0009755	hormone-mediated signaling pathway	109	17	0.00452
BP	GO:0010038	response to metal ion	79	7	0.00545
BP	GO:0090501	RNA phosphodiester bond hydrolysis	57	10	0.00616
BP	GO:1901605	alpha-amino acid metabolic process	171	17	0.00664
BP	GO:0016485	protein processing	31	6	0.0103
BP	GO:0009651	response to salt stress	94	10	0.01211
BP	GO:0009873	ethylene-activated signaling pathway	11	3	0.01261
BP	GO:0072530	purine-containing compound transmembrane transport	11	3	0.01261
BP	GO:1901607	alpha-amino acid biosynthetic process	108	11	0.01327
BP	GO:0023014	signal transduction by protein phosphorylation	34	5	0.01975
BP	GO:0010118	stomatal movement	23	4	0.02052
BP	GO:0006368	transcription elongation from RNA polymerase II promoter	14	3	0.02506
BP	GO:0006665	sphingolipid metabolic process	22	3	0.02877
BP	GO:0009226	nucleotide-sugar biosynthetic process	15	3	0.03027
BP	GO:0009742	brassinosteroid mediated signaling pathway	15	3	0.03027
BP	GO:0006075	(1->3)-beta-D-glucan biosynthetic process	15	3	0.03027
BP	GO:0009658	chloroplast organization	38	5	0.03062
BP	GO:0006796	phosphate-containing compound metabolic process	1699	91	0.03335
BP	GO:0006887	exocytosis	27	5	0.03574
BP	GO:0032147	activation of protein kinase activity	16	3	0.03599
BP	GO:0048827	phyllome development	74	8	0.03869
BP	GO:0045860	positive regulation of protein kinase activity	23	5	0.03877
BP	GO:0090502	RNA phosphodiester bond hydrolysis, endonucleolytic	28	4	0.03954
BP	GO:0006479	protein methylation	39	6	0.04184
BP	GO:0005976	polysaccharide metabolic process	264	17	0.04635
BP	GO:0006476	protein deacetylation	14	3	0.04656
BP	GO:0019941	modification-dependent protein catabolic process	180	10	0.04663
BP	GO:0009991	response to extracellular stimulus	31	2	0.04672
BP	GO:0098754	detoxification	126	5	0.04685
BP	GO:0098660	inorganic ion transmembrane transport	209	8	0.04693
BP	GO:0044267	cellular protein metabolic process	2094	111	0.04769
CC	GO:0000139	Golgi membrane	84	11	0.0029
CC	GO:0000145	Exocyst	10	3	0.0114
CC	GO:0016592	mediator complex	19	4	0.013
CC	GO:0019867	outer membrane	42	5	0.0224
CC	GO:1902554	serine/threonine protein kinase complex	14	3	0.0298
CC	GO:0000148	1,3-beta-D-glucan synthase complex	15	3	0.0359

CC	GO:0019005	SCF ubiquitin ligase complex	15	3	0.0359
MF	GO:0003951	NAD+ kinase activity	10	5	3.50E-05
MF	GO:0016891	endoribonuclease activity, producing 5'-phosphomonoesters	23	5	0.0029
MF	GO:0016791	phosphatase activity	203	18	0.0041
MF	GO:0008375	acetylglucosaminyltransferase activity	17	4	0.0057
MF	GO:0004252	serine-type endopeptidase activity	52	7	0.0076
MF	GO:0005338	nucleotide-sugar transmembrane transporter activity	15	3	0.0111
MF	GO:0003682	chromatin binding	33	6	0.0146
MF	GO:0042803	protein homodimerization activity	35	5	0.0181
MF	GO:0008276	protein methyltransferase activity	29	5	0.0217
MF	GO:0016646	oxidoreductase activity, acting on the CH-NH group of donors, NAD or NADP as acceptor	14	3	0.0218
MF	GO:0004842	ubiquitin-protein transferase activity	123	11	0.0221
MF	GO:0016817	hydrolase activity, acting on acid anhydrides	738	35	0.026
MF	GO:0015923	mannosidase activity	23	3	0.0261
MF	GO:0003843	1,3-beta-D-glucan synthase activity	15	3	0.0264
MF	GO:0051015	actin filament binding	16	3	0.0315
MF	GO:0043169	cation binding	2137	75	0.0342
MF	GO:0016741	transferase activity, transferring one-carbon groups	256	17	0.0439
MF	GO:0033558	protein deacetylase activity	11	3	0.0442
MF	GO:0042626	ATPase activity, coupled to transmembrane movement of substances	178	10	0.0486

Supplementary Table 7 GO enrichment of positively selected genes (PSGs) within *C. gigantea*.

Class	GO.ID	Term	Annotated	PSG	Corrected *P*-value
BP	GO:0051258	protein polymerization	24	4	0.00019
BP	GO:0031327	negative regulation of cellular biosynthetic process	69	4	0.00145
BP	GO:0005983	starch catabolic process	10	3	0.00202
BP	GO:0010468	regulation of gene expression	763	28	0.00684
BP	GO:0080090	regulation of primary metabolic process	831	32	0.00779
BP	GO:0009765	photosynthesis, light harvesting	33	4	0.01143
BP	GO:0006906	vesicle fusion	29	4	0.01163
BP	GO:1901362	organic cyclic compound biosynthetic process	1162	41	0.01919
BP	GO:0015986	ATP synthesis coupled proton transport	23	3	0.02302
BP	GO:0032259	methylation	237	11	0.02537
BP	GO:0009723	response to ethylene	24	3	0.0258
BP	GO:0006378	mRNA polyadenylation	10	2	0.02823
BP	GO:0043269	regulation of ion transport	10	2	0.02823
BP	GO:0097435	supramolecular fiber organization	62	3	0.0283
BP	GO:0006508	proteolysis	515	21	0.03063
BP	GO:0000160	phosphorelay signal transduction system	45	4	0.03241
BP	GO:0051252	regulation of RNA metabolic process	640	23	0.03301
BP	GO:0018130	heterocycle biosynthetic process	1046	37	0.03342
BP	GO:2000377	regulation of reactive oxygen species metabolic process	12	2	0.03997
BP	GO:0006614	SRP-dependent cotranslational protein targeting to membrane	12	2	0.03997
BP	GO:0016117	carotenoid biosynthetic process	12	2	0.03997
BP	GO:0048878	chemical homeostasis	110	4	0.04012
BP	GO:0043623	cellular protein complex assembly	107	11	0.04541
BP	GO:0006563	L-serine metabolic process	25	3	0.04622
BP	GO:0030148	sphingolipid biosynthetic process	13	2	0.04641
CC	GO:0031201	SNARE complex	20	4	0.0028
CC	GO:0009941	chloroplast envelope	159	13	0.003
CC	GO:0009579	thylakoid	203	14	0.0041
CC	GO:0009570	chloroplast stroma	169	11	0.0143
CC	GO:0030135	coated vesicle	40	2	0.0305
CC	GO:0045263	proton-transporting ATP synthase complex, coupling factor F(o)	11	2	0.0425
CC	GO:0005815	microtubule organizing center	11	2	0.0425
MF	GO:0019899	enzyme binding	123	8	0.0021
MF	GO:0000149	SNARE binding	21	3	0.016
MF	GO:0004722	protein serine/threonine phosphatase activity	38	4	0.016
MF	GO:0005543	phospholipid binding	25	3	0.0259
MF	GO:0019239	deaminase activity	10	2	0.026
MF	GO:0005484	SNAP receptor activity	26	3	0.0284
MF	GO:0008170	N-methyltransferase activity	27	3	0.0314
MF	GO:0016868	intramolecular transferase activity, phosphotransferases	12	2	0.0368
MF	GO:0016747	transferase activity, transferring acyl groups other than amino-acyl groups	185	10	0.0389
MF	GO:0061630	ubiquitin protein ligase activity	73	5	0.0398
MF	GO:0016160	amylase activity	13	2	0.0428
MF	GO:0016814	hydrolase activity, acting on carbon-nitrogen (but not peptide) bonds, in cyclic amidines	13	2	0.0428
MF	GO:0016783	sulfurtransferase activity	13	2	0.0428
MF	GO:0043621	protein self-association	14	2	0.0491

Supplementary Table 8 GO enrichment of positively selected genes (PSGs) within *C. duclouxiana* northern population.

Class	GO.ID	Term	Annotated	PSGs	Corrected *P*-value
BP	GO:0043666	regulation of phosphoprotein phosphatase activity	14	3	0.00027
BP	GO:0032269	negative regulation of cellular protein metabolic process	33	3	0.00362
BP	GO:0006188	IMP biosynthetic process	10	2	0.00377
BP	GO:0005977	glycogen metabolic process	12	2	0.00546
BP	GO:0072523	purine-containing compound catabolic process	13	2	0.00642
BP	GO:0009890	negative regulation of biosynthetic process	70	3	0.00931
BP	GO:0045489	pectin biosynthetic process	16	2	0.00969
BP	GO:0006635	fatty acid beta-oxidation	19	2	0.01356
BP	GO:0030162	regulation of proteolysis	40	3	0.01772
BP	GO:0042278	purine nucleoside metabolic process	35	2	0.01864
BP	GO:0006928	movement of cell or subcellular component	56	2	0.01868
BP	GO:0051179	localization	1647	14	0.02878
BP	GO:0043086	negative regulation of catalytic activity	43	3	0.02983
BP	GO:0032535	regulation of cellular component size	26	2	0.04592
BP	GO:0051649	establishment of localization in cell	309	4	0.0462
CC	GO:0005778	peroxisomal membrane	12	2	0.0075
CC	GO:0019005	SCF ubiquitin ligase complex	15	2	0.0116
CC	GO:0005737	cytoplasm	3099	44	0.0201
CC	GO:0015935	small ribosomal subunit	32	2	0.0486
MF	GO:0019888	protein phosphatase regulator activity	12	3	0.0002
MF	GO:0016903	oxidoreductase activity, acting on the aldehyde or oxo group of donors	103	4	0.0015
MF	GO:0048037	cofactor binding	427	10	0.0138
MF	GO:0004185	serine-type carboxypeptidase activity	22	2	0.0198
MF	GO:0016722	oxidoreductase activity, oxidizing metal ions	27	2	0.0291
MF	GO:0016746	transferase activity, transferring acyl groups	263	4	0.0429
MF	GO:0051537	2 iron, 2 sulfur cluster binding	34	2	0.0445

Supplementary Table 9 List of adaptive introgressed genes in *C. duclouxiana* northern population.

Contig ID	Best *Arabidopsis* hit	Gene name	Description
TR68999_c0_g1_i1	AT2G23740.2	*SUVR5*	Histone-lysine N-methyltransferase SUVR5
TR69012_c0_g1_i6	AT1G26090.1		P-loop containing nucleoside triphosphate hydrolases superfamily protein
TR69433_c0_g1_i1	AT3G27325.2		Hydrolases, acting on ester bond
TR70143_c0_g1_i1	AT5G36890.1	*BGLU42*	Beta-glucosidase 42
TR70959_c0_g1_i1	AT2G36885.1		Translation initiation factor
TR75801_c0_g1_i3	AT5G35330.1	*MBD02*	methyl-CPG-binding domain protein 02
TR76070_c0_g1_i2	AT1G72390.1	*PHL*	Nuclear receptor coactivator
TR76111_c0_g1_i2	AT1G70000.1	*MYBD*	Myb-like transcription factor family protein
TR78719_c0_g1_i1	AT4G28706.4		PfkB-like carbohydrate kinase family protein
TR80751_c0_g3_i1	AT1G30500.2	*NF-YA7*	Nuclear factor Y, subunit A7
TR81464_c0_g1_i1	AT1G03530.1	*NAF1*	Nuclear assembly factor 1
TR82065_c0_g1_i1	AT5G05970.2	*NEDD1*	Transducin/WD40 repeat-like superfamily protein
TR85851_c0_g3_i1	AT3G55070.1		LisH/CRA/RING-U-box domains-containing protein
TR87835_c0_g2_i1	AT2G32520.1		Alpha/beta-Hydrolases superfamily protein
TR91797_c0_g2_i1	AT2G18900.1		Transducin/WD40 repeat-like superfamily protein
TR91835_c0_g2_i1	AT2G34357.1		ARM repeat superfamily protein

Supplementary References

1. Excoffier, L., Dupanloup, I., Huerta-Sánchez, E., Sousa, V. C. & Foll, M. Robust demographic inference from genomic and SNP data. *PLoS Genet.* **9**, e1003905 (2013).
